# A novel off-the-shelf solution for the treatment of retrograde false lumen flow causing hemothorax after thoracic endovascular aortic repair for aortic dissection and rupture

**DOI:** 10.1016/j.jvscit.2025.102013

**Published:** 2025-10-15

**Authors:** Jonathan Crisp, Alexander Rolls

**Affiliations:** Vascular Surgery Department, Fiona Stanley Hospital, Perth, Western Australia, Australia

**Keywords:** Aortic dissection, TEVAR, Endovascular

## Abstract

Persistent retrograde false lumen (FL) flow is a common complication following thoracic endovascular aortic repair (TEVAR) for type B aortic dissections. The candy plug technique is a well-established method for FL exclusion; however, device availability can be a limiting factor. We report the case of a 57-year-old man who underwent a novel FL exclusion procedure 2 days post-TEVAR, using a 36 × 66 mm Cook Converter graft and Amplatzer plugs, with complete FL thrombosis and resolution of hemothorax on imaging 7 weeks postoperatively. This off-label technique offers a viable alternative when commercially available candy plug devices are unavailable and urgent intervention is required.

Thoracic endovascular aortic repair (TEVAR) is the first-line surgical approach to treating type B aortic dissections complicated by rupture.^1^ TEVAR promotes false lumen (FL) thrombosis and aortic remodeling, preventing aneurysmal progression. However, complete FL thrombosis only occurs in 40% of cases following entry tear closure with TEVAR,^2^ necessitating further exclusion procedures such as coil embolization or distal extension with branched or fenestrated techniques.

The candy plug (CP) technique is an established method for treating persistent FL perfusion post-TEVAR, with lower rates of incomplete FL thrombosis than other techniques.^3^ Initially described by Kölbel et al,^4^ using a custom-made graft, the technique has evolved, with several commercially available CP devices. These devices are not always available and frequently require patient-specific customization.

This case report presents a patient with worsening left hemithorax following TEVAR for ruptured type B dissection who required urgent FL exclusion with an off-the-shelf device. This was achieved using a converter graft (Cook Medical), combined with Amplatzer plugs (Abbott Medical).

Written informed consent was obtained from the patient for publication of text and associated figures.

## Case report

A hemodynamically unstable 57-year-old male was brought to the emergency department following syncope at home. He had a background of a previous type A aortic dissection extending to the aortic bifurcation, treated with aortic valve resuspension, ascending aorta and arch placement, and a frozen elephant trunk procedure 5 months earlier.

**Fig 1:**
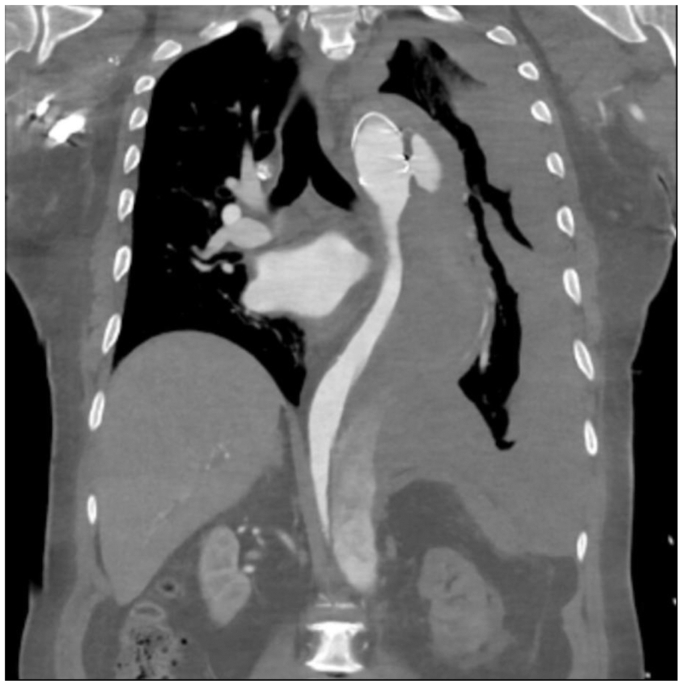
Computed tomography angiography (CTA) on presentation. A coronal image demonstrating a large intramural hematoma in the descending thoracic aorta associated with a left hemothorax.

Computed tomographic angiography (CTA) demonstrated a partially thrombosed descending thoracic FL with a maximum aortic diameter of 80 mm, a large contrast blush at the distal end of the frozen elephant trunk, and a significant hemothorax (Fig 1).

An emergency TEVAR was performed with a 34 × 34 × 202 mm Zenith TX2 endovascular graft (Cook Medical) followed by extension with a barbless 38 × 30 × 202 Zenith TX2 dissection stent (Cook Medical) deployed down to the celiac trunk.

**Fig 2:**
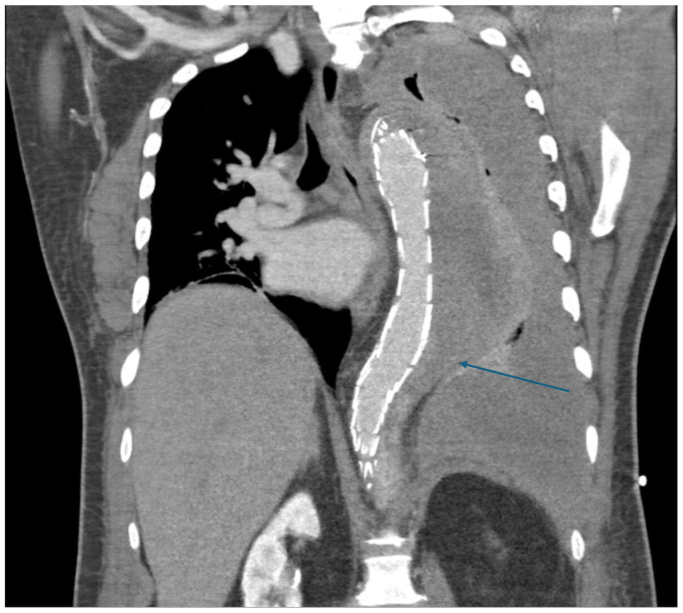
Computed tomography angiography (CTA) following thoracic endovascular aortic repair (TEVAR). A coronal image demonstrating endovascular graft in situ post repair with persistent retrograde flow within the false lumen (FL) and a large left-sided hemothorax. The *blue arrow* indicates the tapering segment of the FL, which was targeted for deployment of the occlusion device.

On postoperative day 1, a chest drain was inserted due to left-sided hemothorax, and on day 2, a repeat CTA demonstrated significant retrograde flow extending from the distal end of the TEVAR into the FL, increasing left hemothorax size and complete collapse of the left lung (Fig 2).

**Fig 3:**
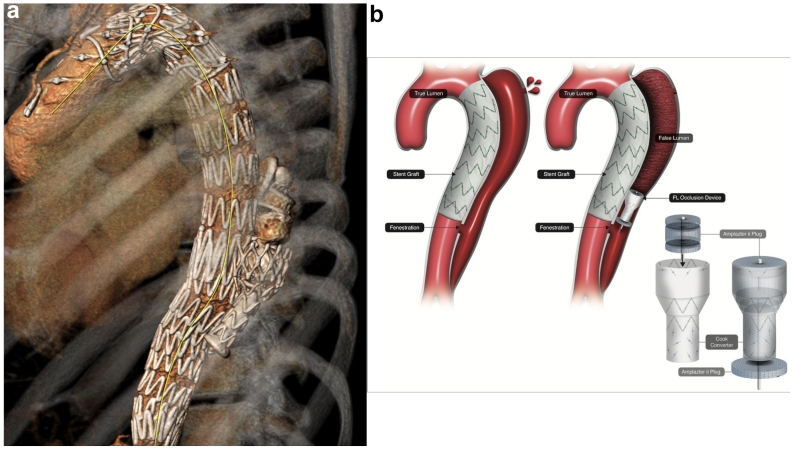
**(A)** Three-dimensional reconstruction of aorta post false lumen (*FL*) exclusion procedure demonstrating Cook converter graft and Amplatzer plugs within FL. **(B)** Schematic diagram of FL exclusion device mechanism.

A decision was made to perform urgent endo-luminal occlusion of the FL; however, commercially available FL occlusion devices were unavailable. The plan was to arrest retrograde flow into the FL by placing a Converter graft (Cook Medical) into the tapered section of the FL, occluding this with Amplatzer plugs (Abbott Medical) (Fig 3, *A* and *B*).

**Fig 4:**
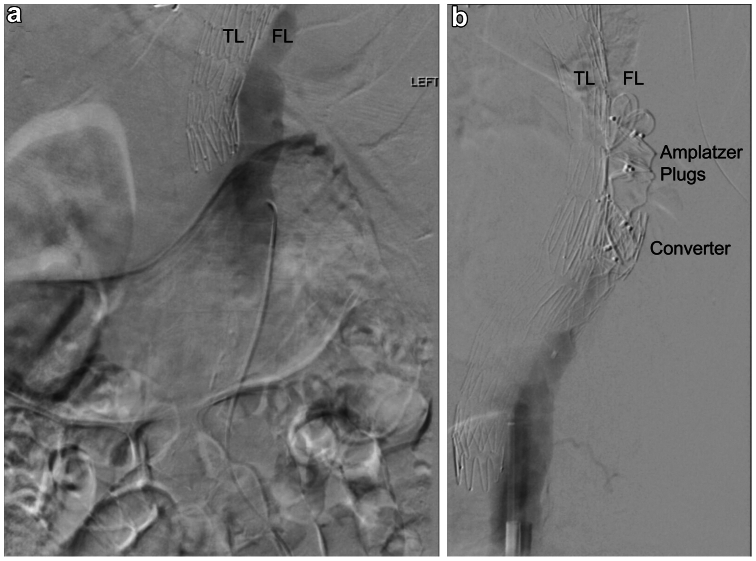
**(A)** Intraoperative angiogram demonstrating persistent retrograde contrast filling of false lumen (*FL*) adjacent to the aortic graft. **(B)** Intraoperative angiogram demonstrating completion runs post FL exclusion procedure with Cook converter graft and Amplatzer plugs preventing contrast filling of FL. *TL*, True lumen.

**Fig 5:**
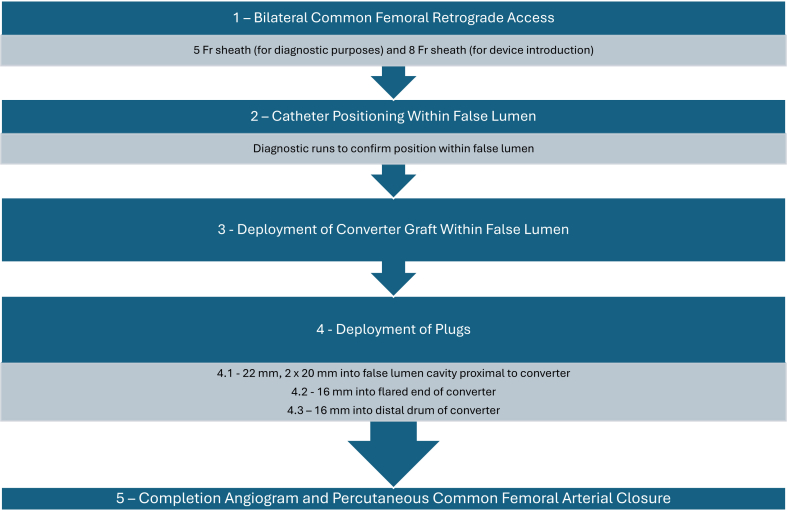
Flow diagram demonstrating procedural steps of modified candy plug (CP) false lumen (FL) exclusion procedure.

Under general anesthesia, ultrasound-guided bilateral common femoral retrograde punctures were performed with a 5 Fr sheath inserted into the right groin for diagnostic purposes and an 8 Fr sheath with 2× ProStyle devices (Abbott Medical) into the left groin, anticipating easier access to the FL from the latter side. Five thousand units of unfractionated heparin were administered as is standard protocol for endovascular techniques within our unit, followed by sequential hand runs, ensuring preferential opacification of the left renal artery (supplied by the FL) as the angled guide-catheter was carefully advanced over a glidewire. Once adjacent to the thoracic stent, a diagnostic pigtail run confirmed position within the FL (Fig 4, *A*). A Lunderquist wire (Cook Medical) was placed in a stable position within the FL up to distal arch. A 36 × 66 mm Converter device (Cook Medical) was advanced over the wire before deployment at a point of natural tapering of the FL, 9.5 cm proximal to the distal end of the TEVAR. Following deployment and delivery system withdrawal, A 7Fr Destination sheath (Terumo) was advanced over the wire, through the converter, and positioned in the large saccular FL. Following this, 22 and 2 × 20 mm Amplatzer II plugs (Abbott Medical) were deployed into the cavity above the converter, filling the FL cavity first while still accessible. An 18-mm Amplatzer II plug was packed into the flared end of the converter. Finally, a 16 mm Amplatzer II plug was deployed such that the distal drum was positioned in the flared section of the converter, the middle drum in the narrow portion, and the proximal drum was allowed to flower out of the bottom of the converter against the walls of the FL, anchoring the construct further. No undue effort was made to densely pack the Amplatzer plugs due to concerns of risk of further rupturing the sac of the FL. There was no concern of compromising true lumen because of the robust TEVAR adjacent to the plugs. Completion angiogram confirmed FL exclusion with no further retrograde filling (Fig 4, *B*) followed by successful ProGlide closure (Fig 5).

**Fig 6:**
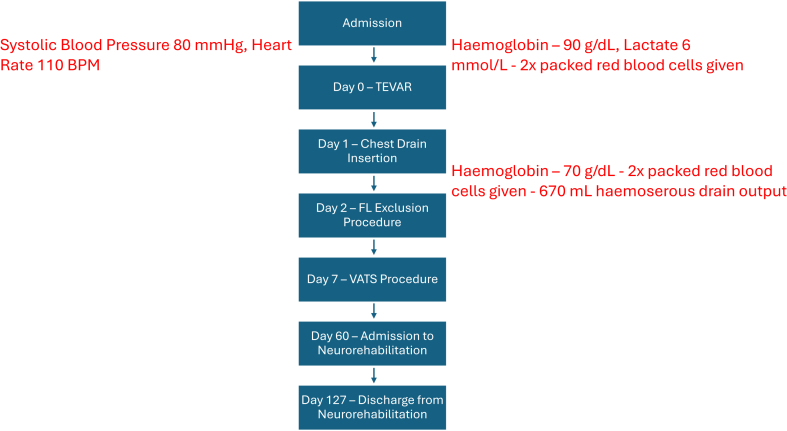
Timeline of events diagram. A diagram of events during admission, referencing day 0 as day of admission and demonstrating hemoglobin trends and drain output prior to false lumen (*FL*) exclusion procedure. *TEVAR*, Thoracic endovascular aortic repair; *VATS*, video-assisted thoracoscopic surgery.

**Fig 7:**
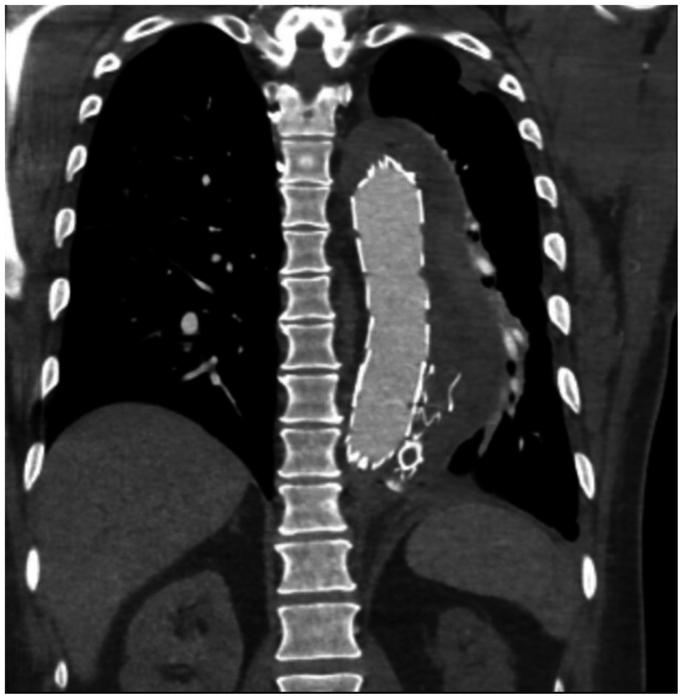
Follow-up computed tomography angiography (CTA) 7 weeks post false lumen (FL) exclusion procedure. A sagittal image demonstrating Cook converter graft and Amplatzer plugs within FL, which has completely thrombosed. The hemothorax has resolved.

Post procedure, he underwent video-assisted left hemithorax evacuation of hematoma after CTA confirmed no further intrathoracic bleeding. Upon extubation and desedation, he was noted to have a persistently reduced Glasgow Coma Scale (GCS), focal right lower limb weakness, and reduced anal tone thought possibly secondary to spinal cord ischemia or an ischemic stroke, and subsequently underwent an urgent rescue spinal drain. A magnetic resonance imaging of the head and spine demonstrated no evidence of spinal cord ischemia; however, it found bifrontal and bioccipital watershed infarcts associated with cortical petechial hemorrhages. He was reviewed by neurology who felt his right lower limb motor deficits were secondary to hypoperfusion injury from watershed infarcts and reduced GCS secondary to hypoxic-ischemic brain injury. After a period in neurorehabilitation, the patient regained baseline speech and mobility before being discharged 4 months post presentation (Fig 6). A 7-week follow-up CTA confirmed FL thrombosis and resolution of hemothorax (Fig 7).

Additional postoperative complications included hyperactive delirium, managed with olanzapine and dexmedetomidine, and clostridium difficle colitis. He experienced a type 2 NSTEMI, hepatic dysfunction, and an acute kidney injury, thought secondary to hypoperfusion from his initial presentation with hemorrhagic shock.

## Discussion

Persistent FL retrograde flow after TEVAR remains a significant challenge, associated with aneurysmal progression and rupture. CP techniques are well-established for achieving FL occlusion,^1^ with low morbidity and mortality rates post intervention.^5^, ^6^, ^7^ Cook Medical have produced several generations of custom-made CP devices available to treat persistent FL disease. The unavailability of CP devices necessitated an off-the-shelf solution with available devices to successfully exclude the patients FL and achieve a satisfactory outcome.

Although there are several reports of modified CP techniques for FL embolization,^8^, ^9^, ^10^ we found no cases in the literature that utilized Cook converter grafts and Amplatzer plugs in the treatment of persistent FL in acute type B aortic dissections. The technique described provides an innovative approach to FL exclusion when CP devices are unavailable, particularly in clinically urgent scenarios associated with aneurysmal progression or hemorrhage. The tapered design of the converter provided an ideal opportunity to interpose fabric between the retrograde inflow into the FL and the rupture site at the distal arch, while narrowing down the channel of flow to 12 mm, which was easily plugged with Amplatzers. Additionally, the converter functioned as a framing device to stabilize the position of the first 16-mm Amplatzer plug, particularly given the extrusion of the proximal drum out of the narrow section.

The off-label nature of this approach introduces important considerations. The deployment of the stent graft must be meticulously planned to obtain a seal. In this case, a favorable position in the mid-descending thoracic aorta was identified where the FL tapered to its narrowest point. This required measuring the FL accurately and selecting an appropriately sized converter. Additional consideration must be made with this technique if a residual endoleak were to be present on completion runs intraoperatively. Options could include extending distally with an off-the-shelf branched device, physician-modified device, or an open thoraco-abdominal aortic repair, which it is unlikely the patient would have tolerated due to his physiological instability. Long-term durability of this technique remains uncertain, especially regarding incomplete thrombosis, persistent retrograde FL flow, and potential plug migration, which could lead to greater aortic growth by comparison to complete thrombosis or complete patency.^11^ However, migration risk has hopefully been mitigated by placing Amplatzer plugs immediately above the converter and stabilization by thrombosis with time.

We propose this technique is most appropriate in the following situations: presence of uncontrolled hemorrhage, insufficient time to manufacture and deliver a custom-made device or incompatibility with available off-the-shelf branched devices, and absence of a single discrete distal descending thoracic fenestration that could be covered with TEVAR extension. Specific follow-up recommendations post procedure include surveillance CTAs at 6 weeks, 6 months, and then 12 months, dependent on findings, and strict blood pressure management aimed at maintaining normotension.

Although this case resulted in a successful outcome in terms of hemorrhage control, its status as a single case report raises questions about its generalizability and reproducibility. Nonetheless, it serves as a valuable point for consideration: over-reliance on one specific device, particularly in resource-limited settings or acute scenarios requiring urgent intervention, leads to delays in providing lifesaving care and adverse patient outcomes. The case serves as a reminder that all endovascular solutions need to be considered broadly when conventional devices are unavailable. Long-term follow-up of this case is warranted to assess durability and monitor for any complications.

## Funding

None.

## Disclosures

None.
